# Effect of Cycloplegia on Keratometric and Biometric Parameters in Keratoconus

**DOI:** 10.1155/2016/3437125

**Published:** 2016-12-12

**Authors:** Nihat Polat, Abuzer Gunduz

**Affiliations:** Department of Ophthalmology, Medical Faculty, Inönü University, Malatya, Turkey

## Abstract

*Purpose.* To obtain information about effect of cycloplegia on keratometry and biometry in keratoconus.* Methods.* 48 keratoconus (Group 1) and 52 healthy subjects (Group 2) were included in the study. We measured the flat meridian of the anterior corneal surface (K1), steep meridian of the anterior corneal surface (K2), lens thickness (LT), anterior chamber depth (ACD), and axial length (AL) using the Lenstar LS 900 before and after cycloplegia.* Results.* The median K1 in Group 1 was 45.64 D before and 45.42 D after cycloplegia, and the difference was statistically significant (*P* < 0.05). The median K2 in Group 1 was 50.96 D before and 50.17 D after cycloplegia, and the difference was significant (*P* < 0.05). The median K1 and K2 in Group 2 were 42.84 and 44.49 D, respectively, before cycloplegia, and 42.84 and 44.56 D after cycloplegia, and the differences were not statistically significant (all *P* > 0.05). There were significant differences in SE, LT, ACD, and RLP between before and after cycloplegia in either Group 1 (all *P* < 0.05) or Group 2 (all *P* < 0.05). There were not statistically significant differences in AL between before cycloplegia and after cycloplegia in either Group 1 (*P* = 0.533) or group 2 (*P* = 0.529).* Conclusions.* Flattened corneal curvature and increase in ACD following cycloplegia in keratoconus patients were detected.

## 1. Introduction

Keratoconus is an ectatic corneal disease characterized by corneal protrusion, irregular astigmatism, and decreased visual acuity due to progressive corneal thinning [1]. Keratoconus leads to biomechanical changes in the cornea, and its definite cause is unknown. The biomechanical features of the cornea are determined by its collagen structure, composition, and collagen fibril bonds. Corneal resistance is primarily determined by the three-dimensional configuration of the collagen lamellae [2]. The corneal collagen structure and organization changes, the extracellular matrix alteration, and keratocyte apoptosis are the main factors in keratoconus that cause corneal biomechanical weakness [3, 4].

Accommodation is the adjustment of the eye's dioptric power to produce a clear retinal image when looking at objects at various distances [5]. The ciliary muscles, zonular fibrils, lens capsule, and lens substance make up the functional accommodation unit [6]. The contraction of the ciliary muscles during accommodation leads to relaxation of the zonular fibrils attached to the crystalline lens equator, resulting in a change in the shape and thickness of the lens [5, 7]. Generally keratoconus is diagnosed at young age, and visual complaints in this age group stand out. Functional accommodation unit of young people works full capacity, and we believe that tonic accommodation cannot be ignored when we evaluated visual problems of keratoconus patients. For this purpose we investigated cycloplegic and noncycloplegic changes to get information about tonic accommodation of keratoconus patients.

Measurement of accommodative biometric changes during tonic accommodation in phakic eyes will provide information about the ways in which the eye responds to tonic accommodation. As far as we are aware, the only study on accommodation in keratoconus patients was conducted in 1990 by Ohmi et al. [8]. Their study included a limited number of subjects and reported accommodative deficiency in keratoconus patients.

Our aim in this study was to obtain information about effect of tonic accommodation on keratometric and biometric measurements in keratoconus patients with and without cycloplegia by using an optical low coherence reflectometer (Lenstar LS 900).

## 2. Materials and Methods

This prospective study was conducted at the ophthalmology department of the Inonu University Faculty of Medicine. The study was planned according to the Helsinki Declaration and after the permission of the local Ethics Committee was received (Reference Number: 2015/91). The patients provided informed written consent. One eye of each patient was included in the study randomly. The study group (Group 1) included one eye each from 48 subjects diagnosed with keratoconus, for a total of 48 eyes. The control group (Group 2) consisted of the 52 eyes of 52 healthy age- and sex-matched subjects who had come to our clinic for refraction. All patients in both groups underwent a standard eye examination by the same ophthalmologist. This examination included refraction biomicroscopic cornea and anterior segment evaluation, fundus examination, and intraocular pressure measurement. The keratoconus diagnosis was made with the classic corneal biomicroscopic findings and the use of the Collaborative Longitudinal Evaluation of Keratoconus (CLEK) Study criteria to evaluate the topographical findings [1, 9, 10]. Eyes with no other problems during the examination were included in the study. The exclusion criteria were a history of corneal or intraocular surgery, a history of contact lens use, a history of ocular trauma, an ocular allergy or dry eye symptoms, the presence of a corneal scar, current pregnancy and/or nursing, and diabetes or a collagen tissue disease. Refractive measurements of the patients were done with an auto kerato-refractometer (KR-8900; Topcon Co., Tokyo, Japan). Keratometric and biometric measurements of the patients were done with the Lenstar LS 900 (Haag Streit AG, Koeniz, Switzerland). Cycloplegia was achieved with 1% cyclopentolate hydrochloride drops administered two times at 10-minute intervals. Cycloplegic refraction examination was performed 45 minutes after the last drop and repeat measurements were taken with the Lenstar LS 900.

### 2.1. Lenstar Measurement

We first focused and aligned the Lenstar using the eye's image on the computer monitor. The subject fixated on an internal fixation light, and we then asked the subject to blink before the measurements. The Lenstar takes 16 consecutive scans per measurement. We obtained 5 measurements (about 20 seconds each) per subject as recommended by the manufacturer. The Lenstar software was used to calculate the mean values. We recorded the flat meridian of the anterior corneal surface (K1), steep meridian of the anterior corneal surface (K2), spherical equivalent (SE), lens thickness (LT), anterior chamber depth (ACD), and axial length (AL) results. The relative lens position (RLP) values were manually recorded following calculation with the following formula: (LT/2 + ACD)/AL.

### 2.2. Statistical Analysis

The IBM SPSS statistical software version 22.0 for Windows was used for statistical analyses. The Shapiro-Wilk test was used to assess normality. All RPL values in both groups conformed to a normal distribution (*P* > 0.05), while the other variables did not (*P* < 0.05). The variables that conformed to a normal distribution were presented as mean ± SD and the ones that did not as median (min-max). The Wilcoxon *t*-test and paired *t*-test were used for intragroup comparisons, while Student's *t*-test and the Mann–Whitney *U* test were used for intergroup comparisons. A *P* value <0.05 was considered significant.

## 3. Results

The age distribution was 24 (12–35) years in Group 1 and 24 (13–35) years in Group 2. Group 1 consisted of 25 female and 23 male subjects, and Group 2 consisted of 27 female and 25 male subjects. The eye distribution was 23 right and 25 left eyes in Group 1 and 26 right and 26 left eyes in Group 2.

**Figure 1:**
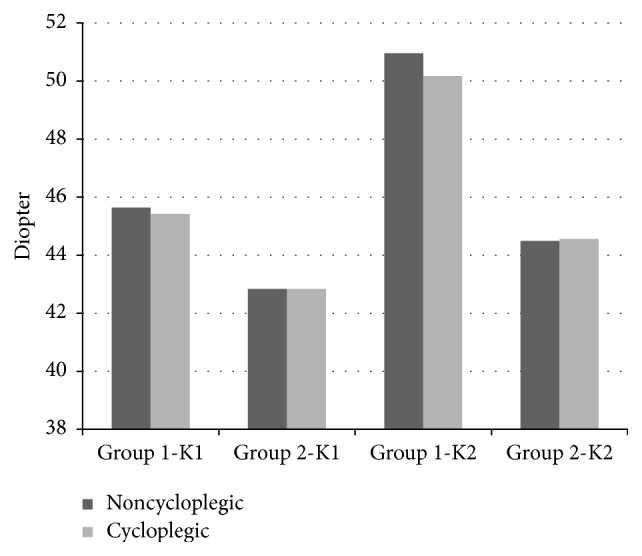
Keratometric changes with cycloplegia. K1: flat meridian of the anterior corneal surface, K2: steep meridian of the anterior corneal surface.

**Figure 2:**
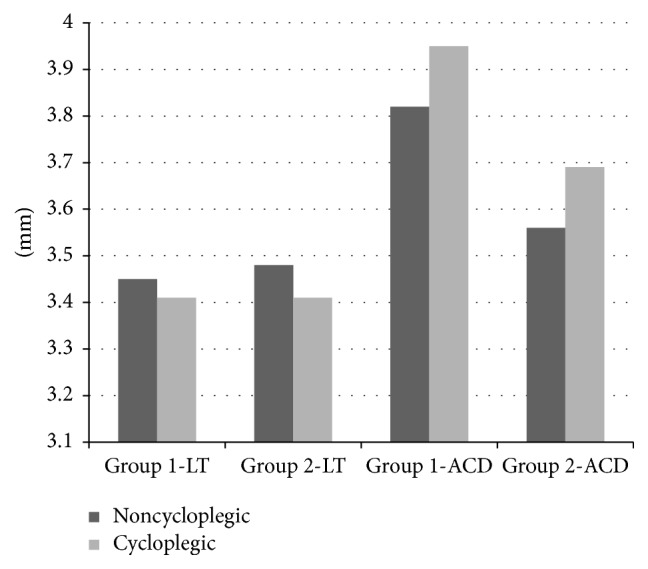
LT and ACD changes with cycloplegia. LT: lens thickness, ACD: anterior chamber depth.

**Figure 3:**
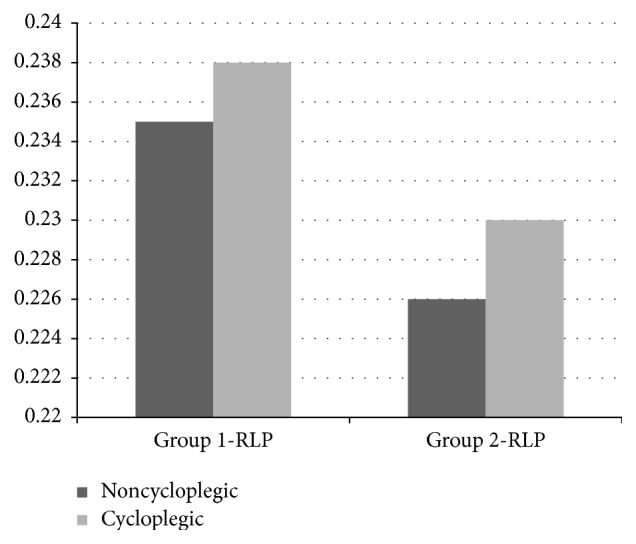
RLP changes with cycloplegia. RLP: relative lens position.

Comparisons of before cycloplegia versus after cycloplegia keratometric parameters, including SE, LT, ACD, AL, and RLP, are presented in Tables 1 and 2. The median K1 before cycloplegia and after cycloplegia in Group 1 was 45.64 and 45.42 D, respectively, and the difference was statistically significant (*P* = 0.017). The median K1 in Group 2 was 42.84 D before cycloplegia and 42.84 D after cycloplegia, and the difference was not significant (*P* = 0.363). The median K2 before cycloplegia and after cycloplegia in Group 1 was 50.96 and 50.17 D, respectively, and the difference was significant (*P* = 0.001). The median K2 in Group 2 was 44.49 D before cycloplegia and 44.56 D after cycloplegia and the difference was not significant (*P* = 0.660) (Figure 1). There were significant differences in SE, LT, ACD (Figure 2), and RLP (Figure 3) between before cycloplegia and after cycloplegia in both Group 1 (all *P* < 0.001) and Group 2 (all *P* < 0.001). There were not statistically significant differences in AL between before cycloplegia and after cycloplegia in either Group 1 (*P* = 0.533) and Group 2 (*P* = 0.529).

A significant difference was present in terms of the K1, K2, SE, ACD, and RLP measurements in both the noncycloplegic and cycloplegic states in intergroup comparisons (all *P* < 0.001). No significant difference was present in terms of the LT or AL in the noncycloplegic and cycloplegic states (*P* = 0.280, *P* = 0.357, resp., and *P* = 0.503, *P* = 0.506, resp.) (Tables 3 and 4).

## 4. Discussion

As far as we are aware, our study is the first to evaluate the effects of cycloplegia on ocular biometry measurements and lens parameters in keratoconus patients.

Studies on the corneal effect of accommodation have produced contrasting results. Some studies have found corneal steepening with ciliary contraction and corneal flattening with cycloplegia [11, 12] while others have not found such an effect [13, 14]. Studies on myopic eyes have revealed corneal flattening with cycloplegia [12, 15, 16]. Some of the above studies have been performed in children and some on myopic eyes; we therefore believe that the low ocular tissue rigidity in the patient groups of these studies is responsible for the results. As far as we are aware, there is no study on the effect of accommodation on the cornea in keratoconus patients. The biomechanically weak cornea in keratoconus patients suggests that it may be influenced by the contraction of the adjacent ciliary muscles. Our results demonstrated that the K1 and K2 values showed a statistically significant decrease following cycloplegia in the keratoconus group, meaning that the cornea had become flatter. We believe that the relaxation in the ciliary muscles following cycloplegia could be responsible for the decrease in K1 and K2. We did not see such an effect in the control group, possibly because the cornea is biomechanically more stable. This change in corneal curvature in keratoconus patients due to the movement of ciliary muscles leads to a continuous change in K values and concomitant accommodation in various degrees during the day, causing constantly changing refractive values. We feel that this factor could be contributing to the constantly changing visual fluctuations in keratoconus patients and the difficulty in fitting contact lenses in some of these patients.

The significant difference in SE values both within and between groups indicates the presence of an effective accommodation in the patients. A blocked accommodation and the resultant decrease in lens power have historically been thought to be the reason for the postcycloplegia refractive changes. However, it is possible that the corneal power, ACD, and AL changes that are also present in this state influence the refractive state following cycloplegia [12]. This can be explained as the ACD changes are likely due to lens changes and the changes in corneal power can be calculated from the changes in K readings before and after cycloplegia. Our results show that cycloplegia has no significant effect on the AL in the keratoconus or the control group. There is no previous study on the effect of cycloplegia in keratoconus patients, but other studies on various patient groups stated that cycloplegia has no significant effect on the AL [17–19].

An increase in the ACD in keratoconus patients is widely recognized when compared with the age-matched control subjects. Emre et al. [20] found that the ACD showed a significant increase with an increase in the keratoconus stage and that this increase could be due to anterior protrusion of the cornea. Our results also indicated that the ACD values in keratoconus patients were significantly higher than in the control group. We found increased ACD values in keratoconus patients when the accommodative effect was eliminated with cycloplegia, and the control group showed a similar change. The increased ACD following cycloplegia is due to the backwards movement and flattening of the lens [15, 17]. The lack of any change in the AL with cycloplegia supports the notion that the ACD increase originates from the lens. The effect of accommodation on the ACD has been demonstrated in many studies. These studies have reported an increase in the ACD following the elimination of accommodation with cycloplegia [17, 19]. Our results also indicate that such a relationship between accommodation and the ACD continues in keratoconus patients. These changes are important, as the ACD value is used in biometric formulae such as Haigis and Holladay 2 and for phakic intraocular lens (IOL) insertion. Studies that have measured ACD changes following the stimulation of accommodation have found lower ACD values with increasing accommodation [21–24]. There are no studies on stimulated accommodation in keratoconus patients. We also did not prefer accommodation stimulation in our study, and this may be one of our limitations. It is known that increased lens thickness following accommodation decreases the ACD. The increased lens thickness is the result of a forward advancement of the lens anterior surface, but there are various reports about the backward displacement of the lens posterior surface on lens thickness [25]. Monkey studies have shown that 75% of the increased lens thickness is due to anterior advancement of the anterior surface, while 25% is due to posterior displacement of the posterior surface [26, 27]. We found that noncycloplegic and cycloplegic lens thickness was similar between the keratoconus patients and the control group. Ernst and Hsu [28] have reported a lens thickness of 3.92 ± 0.42 mm in KC patients and 4.03 ± 0.40 mm in the emmetrope group using immersion-ultrasound biometry, which is not a statistically significant difference. However, their lens thickness results were much higher than ours. We believe this difference is due to the age of their subjects because their subjects are older than our subjects. Also this difference may be due to the measurement device used. Another study comparing ultrasound biometry with the Lenstar has reported results that support this theory [29]. We found a decrease in the lens thickness following cycloplegia in the keratoconus group, and the control group also showed a decrease. The lack of a significant difference between the lens thicknesses of the keratoconus and control groups following cycloplegia also indicates a similar degree of response to cycloplegia in the two groups.

We also calculated the RLP in our study. It has been stated that the RLP can provide an idea as to the ciliary process location [30]. Our noncycloplegic RLP results indicated that the lens was 0.09 units more posterior in the KC group than in the control group. We believe this difference was due to the difference in the ACD, as there was no change in the other variables (LT, AL) used in the RLP calculation. The cycloplegic RLP measurement was found to increase similarly in both groups, indicating a posterior movement of the lens center. The difference between the cycloplegic and noncycloplegic RLP was similar in both groups, and so the posterior movement was similar in the two groups. The similar RLP change indicates similar functional responses to cycloplegia in both the keratoconus and control groups. A recently published study reported that the use of mini scleral lens causes impaired accommodative response in keratoconus patients. The authors speculated that it may be associated with microstructural changes in the posterior chamber caused by scleral lenses resting on the bulbar conjunctiva and sclera [31]. Our results are in accordance with this view. Weak accommodation in myopic eyes has been reported in previous studies [32]. There are also case reports on lens disorders in keratoconus [33]. We found that keratoconus patients had myopic refractive values, but physiological accommodation resulted in effective lens thicknesses and refractive results (a difference in the SE) as in the control group. The similar lens thicknesses, thickness changes, and lens movement amounts indicate normal accommodative function in keratoconus patients.

Many studies on the change in biometric parameters and lens parameters with accommodation have used low resolution measurement devices or subjective methods, leading to a wide range of results [34]. Most of these studies have also stimulated accommodation, again causing variable results [35]. Taking measurements from the other eye in such studies after stimulating accommodation makes the results suspect [34]. We obtained measurements following the physiological accommodation stimulated by the device and then evaluated changes in the parameters of the same eye following cycloplegia in our study. Our measurements were taken with a noncontact optical low coherence reflectometer that has proven accuracy and reliability [36, 37].

We believe our study could guide others in studies on the keratometric, biometric, and lenticular changes in keratoconus patients, as well as in obtaining more exact and desired results from the refractive surgery procedures (such as phakic IOL insertion) that may be required in such patients. We also believe that this information may be useful for refractive examination and contact lens application, which are problematic in keratoconus patients.

In conclusion, we detected a flattened corneal curvature, a positive shift in SE, and an increase in the ACD following cycloplegia in keratoconus patients. The decrease in lens thickness and backward movement of the lens were at significant levels. There was no significant change in the AL. Our results indicate that the crystalline lens is relatively more posterior in keratoconus patients, but the ciliary process offered a normal response to the accommodation-cycloplegia process.

## Figures and Tables

**Table 1:** Response to cycloplegia in Group 1 (*n* = 48).

	Before cycloplegia	After cycloplegia	*P*
K1 (D)	45.64 (42.06–57.74)	45.42 (41.99–58.61)	0.017^*∗*^
K2 (D)	50.96 (44.52–62.86)	50.17 (44.39–64.41)	0.001^*∗*^
SE (D)	−4.05 (0.75–17.85)	−3.25 (0.65–14.85)	0.0001^*∗*^
LT (mm)	3.45 (3.10–4.04)	3.41 (3.01–3.94)	0.0001^*∗*^
ACD (mm)	3.82 (3.44–4.22)	3.95 (3.47–4.31)	0.0001^*∗*^
AL (mm)	23.58 (21.91–25.55)	23.56 (21.96–25.51)	0.533
RLP (mean ± SD)	0.235 ± 0.01	0.238 ± 0.01	0.0001^*∗*^

K1: flat meridian of the anterior corneal surface, K2: steep meridian of the anterior corneal surface, SE: spherical equivalent, LT: lens thickness, ACD: anterior chamber depth, AL: axial length, RLP: relative lens position, D: diopter, and (−): negative value. ^*∗*^Statisticaly significant.

**Table 2:** Response to cycloplegia in Group 2 (*n* = 52).

	Before cycloplegia	After cycloplegia	*P*
K1 (D)	42.84 (38.87–47.03)	42.84 (38.53–47.01)	0.363
K2 (D)	44.49 (40.61–52.52)	44.56 (40.14–52.61)	0.660
SE (D)	−1.5 (0.25–7.60)	−0.75 (0–7.50)	0.0001^*∗*^
LT (mm)	3.48 (3.21–4.10)	3.41 (3.12–4.02)	0.0001^*∗*^
ACD (mm)	3.56 (2.80–4.39)	3.69 (3.10–4.46)	0.0001^*∗*^
AL (mm)	23.57 (22.05–27.30)	23.58 (22.03–27.28)	0.529
RLP (mean ± SD)	0.226 ± 0.01	0.230 ± 0.01	0.0001^*∗*^

K1: flat meridian of the anterior corneal surface, K2: steep meridian of the anterior corneal surface, SE: spherical equivalent, LT: lens thickness, ACD: anterior chamber depth, AL: axial length, RLP: relative lens position, D: diopter, and (−): negative value. ^*∗*^Statisticaly significant.

**Table 3:** Noncycloplegic situation between groups.

Variables	Group 1	Group 2	*P*
K1 (D)	45.64 (42.06–57.74)	42.84 (38.87–47.03)	0.0001^*∗*^
K2 (D)	50.96 (44.52–62.86)	44.49 (40.61–52.52)	0.0001^*∗*^
SE (D)	4.05 (0.75–17.85)	1.5 (0.25–7.60)	0.0001^*∗*^
LT (mm)	3.45 (3.10–4.04)	3.48 (3.21–4.10)	0.280
ACD (mm)	3.82 (3.44–4.22)	3.56 (2.80–4.39)	0.0001^*∗*^
AL (mm)	23.58 (21.91–25.55)	23.57 (22.05–27.30)	0.503
RLP (mean ± SD)	0.235 ± 0.01	0.226 ± 0.01	0.0001^*∗*^

K1: flat meridian of the anterior corneal surface, K2: steep meridian of the anterior corneal surface, SE: spherical equivalent, LT: lens thickness, ACD: anterior chamber depth, AL: axial length, RLP: relative lens position, and D: diopter. ^*∗*^Statisticaly significant.

**Table 4:** Cycloplegic situation between groups.

Variables	Group 1	Group 2	*P*
K1 (D)	45.42 (41.99–58.61)	42.84 (38.53–47.01)	0.0001^*∗*^
K2 (D)	50.17 (44.39–64.41)	44.56 (40.14–52.61)	0.0001^*∗*^
SE (D)	3.25 (0.65–14.85)	0.75 (0–7.50)	0.0001^*∗*^
LT (mm)	3.41 (3.01–3.94)	3.41 (3.12–4.02)	0.357
ACD (mm)	3.95 (3.47–4.31)	3.69 (3.10–4.46)	0.001^*∗*^
AL (mm)	23.56 (21.96–25.51)	23.58 (22.03–27.28)	0.506
RLP (mean ± SD)	0.238 ± 0.01	0.230 ± 0.01	0.0001^*∗*^

K1: flat meridian of the anterior corneal surface, K2: steep meridian of the anterior corneal surface, SE: spherical equivalent, LT: lens thickness, ACD: anterior chamber depth, AL: axial length, RLP: relative lens position, and D: diopter. ^*∗*^Statisticaly significant.
